# Simultaneous versus staged approach in transcatheter aortic valve implantation for severe stenosis and endovascular aortic repair for thoracic and abdominal aortic aneurysm

**DOI:** 10.1093/ejcts/ezae379

**Published:** 2024-10-23

**Authors:** Enrico Gallitto, Paolo Spath, Gian Luca Faggioli, Francesco Saia, Tullio Palmerini, Michele Piazza, Mario D’Oria, Gioele Simonte, Antonio Cappiello, Giacomo Isernia, Guido Gelpi, Antonio Rizza, Gabriele Piffaretti, Mauro Gargiulo, the Italian Multicenter T/EVAR + TAVI Study’s Group, M Antonello, R Bellosta, S Berti, A Bramucci, A Cappiello, F Cecere, L Di Marzo, M D’Oria, G L Faggioli, A Freyrie, E Gallitto, M Gargiulo, G Gelpi, S Gennai, G Isernia, S Lepidi, M Lodato, C Marrozzini, T Palmerini, G Pratesi, M Piazza, W Mansour, L Mezzetto, G Piffaretti, A Rizza, F Saia, R Silingardi, G Simonte, F Squizzato, P Spath, G Tinelli, M Tozzi, S Trimarchi, G F Veraldi

**Affiliations:** Vascular Surgery, University of Bologna-DIMEC, Bologna, Italy; Vascular Surgery unit, IRCCS Azienda Ospedaliero-universitaria di Bologna; Vascular Surgery, University of Bologna-DIMEC, Bologna, Italy; Vascular Surgery unit, IRCCS Azienda Ospedaliero-universitaria di Bologna; Vascular Surgery, University of Bologna-DIMEC, Bologna, Italy; Vascular Surgery unit, IRCCS Azienda Ospedaliero-universitaria di Bologna; Interventional Cardiology, IRCCS Azienda Ospedaliero-universitaria di Bologna; Interventional Cardiology, IRCCS Azienda Ospedaliero-universitaria di Bologna; Vascular Surgery, University of Padova, Padova, Italy; Vascular Surgery, University of Trieste, Trieste, Italy; Vascular and Endovascular Surgery Unit, Hospital S. Maria Misericordia, Perugia, Italy; Vascular Surgery, University of Bologna-DIMEC, Bologna, Italy; Vascular and Endovascular Surgery Unit, Hospital S. Maria Misericordia, Perugia, Italy; Cardiac Surgery, IRCCS Ca’ Granda, Ospedale Maggiore Policlinico, Milan, Italy; Cardiology Unit, Fondazione Toscana Gabriele Monasterio, Massa, Italy; Vascular Surgery, Department of Medicine and Surgery, University of Insubria School of Medicine, ASST-Settelaghi Universitary Teaching Hospital, Varese, Italy; Vascular Surgery, University of Bologna-DIMEC, Bologna, Italy; Vascular Surgery unit, IRCCS Azienda Ospedaliero-universitaria di Bologna

**Keywords:** Transcatheter aortic valve implantation, Endovascular aortic repair, Abdominal aortic aneurysm, Transcatheter aortic valve implantation, Endovascular aortic repair, Thoracic endovascular aortic repair

## Abstract

**OBJECTIVES:**

Thoracic/abdominal aortic aneurysms and aortic stenosis may be concomitant diseases requiring both transcatheter aortic valve implantation (TAVI) and endovascular aneurysm repair (T/EVAR) in high-risk patients for surgical approaches, but temporal management is not clearly defined yet. The aim of the study was to analyse outcomes of simultaneous versus staged TAVI and T/EVAR.

**METHODS:**

Retrospective observational multicentre study was performed on patients requiring TAVI and T/EVAR from 2016 to 2022. Patients were divided into 2 groups: ‘Simultaneous group’ if T/EVAR + TAVI were performed in the same procedure and ‘Staged group’ if T/EVAR and TAVI were performed in 2 steps, but within 3 months. Primary outcomes were technical success, 30-day mortality/major adverse events and follow-up survival. Secondary outcomes were procedural metrics and length of stay.

**RESULTS:**

Forty-four cases were collected; 8 (18%) had T/EVAR and 36 (82%) had EVAR, respectively. Upon temporal determination, 25 (57%) and 19 (43%) were clustered in Simultaneous and Staged groups, respectively. In Staged group, median time between procedures was 72 (interquartile range—IQR: 57–87) days. Preoperative and intraoperative figures were similar. There was no difference in 30-day mortality (Simultaneous: 0/25 versus Staged: 1/19; *P* = 0.43). Pulmonary events (Simultaneous: 0/25 versus Staged: 5/19; *P* = 0.01) and need of postoperative cardiac pacemaker (Simultaneous: 2/25 versus Staged: 7/19; *P* = 0.02) were more frequent in Staged patients. The overall length of stay was lower in the Simultaneous group [Simultaneous: 7 (IQR: 6–8) versus Staged: 19 (IQR: 15–23) days; *P* = 0.001]. The median follow-up was 25 (IQR: 8–42) months and estimated 3-year survival was 73% with no difference between groups (Simultaneous: 82% versus Staged: 74%; *P* = 0.90).

**CONCLUSIONS:**

Both Simultaneous or Staged T/EVAR and TAVI procedures are effective with satisfactory outcomes. Despite the small numbers, simultaneous repair seems to reduce length of stay and pulmonary complications, maintaining similar follow-up survival.

## Graphical Abstract

**Figure:**
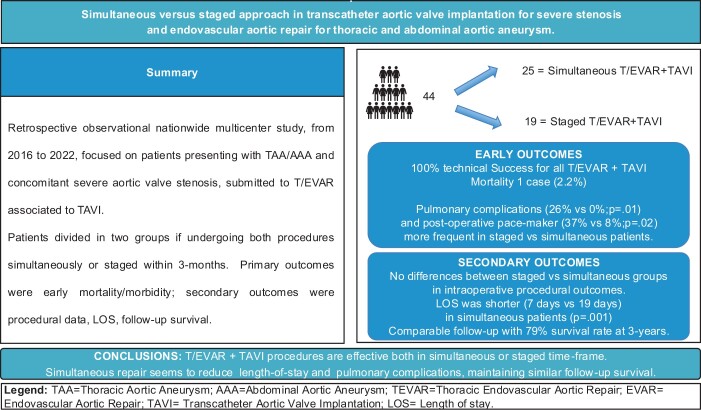

## INTRODUCTION

According to the current international guidelines, transcatheter aortic valve implantation (TAVI) is the recommended option for treating patients with symptomatic and severe aortic valve stenosis (AS) in older patients (≥75 years) and at high-risk or anatomically unsuitable for surgical aortic valve replacement (SAVR). Trans-femoral (TF) approach is an available option with reduced perioperative morbidity and mortality when compared to transaxillary, transaortic and transapical routes [1–3].

The presence of concomitant aortic–iliac arterial diseases or vascular access complications during TF-TAVI may reduce the benefits of this approach as they are associated with prolonged hospitalization and postoperative increased mortality rates [4, 5]. Concomitant AS and thoracic or abdominal aortic aneurysms (TAAs/AAAs) are not uncommon [6], but no clear recommendations are reported into guidelines [1, 7, 8] and their ideal temporal management is yet to be defined since only anecdotal data are available about concomitant endovascular aneurysm repair (T/EVAR) and TF-TAVI [9]. From a hypothetical standpoint, a simultaneous repair may benefit exposing the patient to a single procedure; however, issues might be considered in combining 2 main interventions in the same setting. Therefore, the aim of the study was to report the results of the endovascular management of concomitant severe AS and TAAs or AAAs, both in simultaneous and staged approach.

## METHODS

### Study design/patient selections

It was a retrospective observational, nationwide study focused on patients with concomitant severe and symptomatic AS and presenting with symptomatic/asymptomatic TAAs or AAAs, undergoing TF-TAVI and T/EVAR, between 2016 and 2022.

Patients were divided into 2 groups:

- Simultaneous group: T/EVAR + TF-TAVI in the same procedure.
- Staged group: T/EVAR and TF-TAVI performed within 3 months.

Data from the Simultaneous and Staged groups were compared for the study’s outcomes [10].

### Preoperative work-up

Patients were evaluated for an aortic valve replacement in case of severe and symptomatic AS, confirmed by transthoracic echocardiography (mean gradient >40 mmHg or aortic valve area <1.0 cm^2^) [1]. A multidisciplinary Heart Team, composed of Cardiologists, Interventional Cardiologists, Cardiac Anaesthetists and Cardiac Surgeons, was involved in the patient selection, and older patients (≥75 years) or high surgical risk for surgical aortic valve replacement (SAVR) were considered for TAVI [1]. An ECG-gated cardiac and thoraco-abdominal computed tomography angiography was evaluated for the valve-graft sizing and femoral/iliac or axillary access analysis. Patients were included in the study only if TAVI procedure was performed by transfemoral approach. In case of any vascular issue, an adjunctive preoperative consultation by Vascular Surgeons was performed. Indication for T/EVAR was considered by Vascular Surgeon according to the current guidelines [7, 8]. Patients were decided to undergo prior T/EVAR or TAVI or do both interventions in the same procedures, based on specific patients’ fitness, urgency of the repair per each pathology and institutional protocols. Patients with staged procedures with interval time longer than 3 months were arbitrary excluded from the study in order to reduce confounding factor in this specific fragile population that may interfere with the specific outcomes of the procedures. Aiming to analyse procedural outcomes, patients who did not perform both procedures due to clinical or other issues were excluded from the study.

### Definitions and outcomes

Technical success, 30-day mortality/major adverse events and follow-up survival were assessed as primary outcomes. Procedure/fluoroscopy time, contrast media volume and hospitalization were evaluated as secondary outcomes. The cumulative data/events from both procedures for the Staged group were taken into account when comparing with the Simultaneous group.

Technical success was defined as the combination of successful deployment of the cardiac valve according to the Valve Academic Research Consortium (VARC)-3 definition and aortic endograft [11].

Thirty-day mortality and major adverse events were classified as by reporting standards [11]. Vascular complications were defined and classified according to the VARC 3 guidelines [12].

### Statistical analysis

Continuous data were reported as a median and interquartile range (IQR). Categorical data were expressed as frequency. Differences between Simultaneous versus Staged groups were evaluated by Fisher’s exact test and Mann–Whitney test for categorical and continuous variables. Follow-up survival analysis was estimated by Kaplan–Meier analysis, and difference between Simultaneous versus Staged groups was evaluated by Log-Rank. Univariate analysis were performed and logistic regression multivariate analysis models were used to adjust for confounders. *P* value was considered significant when it was <0.05. Statistical analysis was performed by SPSS 28.0 (SPSS Inc., Chicago, IL, USA).

## RESULTS

### Patients selection

Forty-four patients required concomitant or early deferred aortic aneurysm repair, and TF-TAVI: 8 (18%) had a TAA and 36 (82%) an AAA, respectively. The median age and aneurysm diameter were 82 (IQR: 73–87) years and 58 (IQR: 50–71) mm, respectively. Three (7%) patients had a symptomatic aneurysm with abdominal pain and were treated by standard endovascular infrarenal repair 1st followed by staged TF-TAVI, 5 (11%) had acute heart failure at the moment of hospitalization and 14 (32%) had a history of acute heart failure within 3 preprocedural months. Twenty-five (57%) and 19 (43%) cases were grouped in Simultaneous and Staged groups, respectively. Demographics and preoperative data are reported in Table 1 and they were similar in the 2 groups, except for female gender (*P* = 0.001), more frequent in the Simultaneous group.

**Table 1:** Demographics and preoperative risk-factors

	Overall—44, *N* (%)	Simultaneous—25, *N* (%)	Staged—19, *N* (%)	*P-*value
Male	30 (68)	13 (52)	17 (89)	0.01
Body mass index >31	8 (18)	5 (20)	3 (18)	0.27
Hypertension	41 (93)	24 (96)	17 (89)	0.57
Dyslipidaemia	40 (91)	22 (88)	18 (95)	0.62
Active smoker	9 (20)	5 (20)	4 (21)	0.53
History of smoke	22 (50)	11 (44)	11 (57)	0.28
Diabetes	9 (21)	5 (20)	4 (21)	1
Chronic obstructive pulmonary disease	15 (34)	8 (32)	7 (36)	0.75
Coronary artery disease	27 (61)	13 (52)	14 (74)	0.21
Atrial fibrillation	10 (23)	6 (24)	4 (21)	0.47
Cerebral vascular insufficiency	7 (16)	3 (12)	4 (21)	0.21
Peripheral arterial occlusive disease	8 (18)	5 (20)	3 (16)	1.0
Chronic renal failure	20 (45)	12 (48)	8 (42)	0.13
Dialysis	0 (0)	0 (0)	0 (0)	–
History of heart failure (within 3 months)	14 (32)	9 (36)	5 (26)	0.60
Active heart failure	5 (11)	3 (12)	2 (12)	1
Medical therapy				
Dual antiplatelet	13 (30)	6 (24)	7 (36)	0.57
Anticoagulant therapy	13 (30)	8 (32)	5 (26)	0.74
Statin	42 (96)	23 (92)	19 (100)	0.49
Previous infrarenal aortic repair	7 (16)	3 (12)	4 (21)	0.44
Surgical	3 (7)	1 (4)	2 (12)	0.60
Endovascular	5 (11)	2 (8)	3 (18)	0.63
American Score of Anesthesiologist				
3	14 (32)	6 (24)	8 (42)	0.32
4	30 (68)	19 (76)	11 (57)	0.33
Hostile bilateral femoral/iliac access	11 (25)	6 (24)	5 (26)	0.89
	Median (IQR)	Median (IQR)	Median (IQR)	
Age (years)	82 (78–86)	81 (76–86)	83 (79–87)	0.32
Preoperative creatinine (mg/dl)	1.3 (0.9–1.7)	1.2 (1.0–1.4)	1.3 (1.1–1.5)	0.43
Preoperative eGFR (ml/min)	59 (45–73)	59 (44–73)	58 (45–71)	0.23
Aneurysm diameter (mm)	58 (55–61)	57 (55–59)	61 (57–65)	0.07

IQR: interquartile-range; N: numbers.

### Procedure

Table 2 summarizes the major procedural details. The median time between the T/EVAR and TAVI procedures in the Staged group was 72 (IQR: 57–87) days. Technical success (T/EVAR + TF-TAVI) was achieved in all cases. Details of the endograft used for T/EVAR procedures and type of valves used for TAVI are summarized in Supplementary Material, Table S1.

**Table 2:** Procedural details

	Overall—44, *N* (%)	Simultaneous—25, *N* (%)	Staged—19, *N* (%)	*P-*value
Anaesthesia for TEVAR/EVAR				
Local	16 (34)	9 (36)	7 (37)	1
Loco-regional	5 (11)	2 (8)	3 (16)	0.63
General	23 (52)	14 (56)	9 (47)	0.76
Femoral access TEVAR EVAR				
Percutaneous	27 (61)	16 (64)	11 (58)	0.76
Surgical cut down	17 (39)	9 (36)	8 (42)	0.92
Femoral access TAVI				
Percutaneous	30 (68)	16 (64)	14 (74)	0.28
Surgical cut down	14 (32)	9 (36)	5 (26)	0.63
Aortic endograft configuration				
Tube	7 (16)	3 (12)	4 (21)	0.44
Aortic–bi-iliac	37 (84)	22 (88)	15 (79)	0.44
Iliac artery balloon angioplasty	2 (5)	1 (4)	1 (5)	1
Iliac artery stenting	2 (5)	1 (4)	1 (5)	1
Hypogastric artery embolization	2 (5)	0 (0)	2 (11)	0.10
Blood transfusion	12 (27)	9 (36)	3 (16)	0.18
Technical success	44 (100)	25 (100)	19 (100)	1
Type II endoleak	2 (5)	1 (4)	1 (5)	1
	Median (IQR)	Median (IQR)	Median (IQR)	
Size of main access for T/EVAR (Fr)	18 (16–20)	18 (16–20)	18 (16–20)	1
Size of main access for TAVI (Fr)	14 (13–15)	14 (12–16)	14 (12–16)	1
Procedural time (min)	181 (163–199)	175 (156–194)	190 (179–201)	0.87
Fluoroscopy time (min)	38 (32–446)	40 (36–44)	42 (39–45)	0.90
Contrast media volume (ml)	203 (181–223)	202 (166–238)	205 (187–223)	0.20

EVAR: endovascular aortic repair; Fr: French; IQR: interquartile range; N: Numbers; T/EVAR: endovascular aneurysm repair; TAVI: transcatheter aortic valve implantation; TEVAR: thoracic endovascular aortic repair.

### Early results

Table 3 summarizes adverse events within 30 postoperative days. Pulmonary adverse events (Simultaneous: 0/25 versus Staged: 5/19 versus; *P* = 0.01) and the need of postoperative cardiac pacemaker (Simultaneous: 2/25 versus Staged: 7/19; *P* = 0.02) were more frequent in the Staged group. One (2%) patient died within 30 days (Simultaneous: 0/25 versus Staged: 1/19; *P* = 0.43): an 84 year-old male who underwent EVAR 1st and TF-TAVI after 86 days; the 2nd postoperative course was complicated by urinary sepsis causing final exitus. The overall hospitalization was higher in the Staged group than the Simultaneous one [Simultaneous: 7 (IQR: 6–8) versus Staged: 19 (IQR: 15–23) days; *P* = 0.001].

**Table 3:** Adverse events within 30 postoperative days

	Overall-44, *N* (%)	Simultaneous-5, *N* (%)	Staged-19, *N* (%)	*P*-value
Cardiac adverse events	2 (5)	0 (0)	2 (11)	0.18
Cerebrovascular adverse events	2 (5)	1 (4)	1 (5)	1
Gastrointestinal adverse events	0 (0)	0 (0)	0 (0)	–
Renal function worsening	5 (11)	2 (8)	3 (16)	0.64
Dialysis	0	0	0	–
Pulmonary adverse events	5 (11)	0 (0)	5 (26)	0.01
Need of postoperative cardiac pacemaker	9 (21)	2 (8)	7 (37)	0.02
Reinterventions	1 (2)	0 (0)	1 (5)	0.43
Vascular access complication	2 (5)	1 (4)	1 (5)	1
Death	1 (2)	0 (0)	1 (5)	0.43

Among the 5 cases of renal function worsening reported at 24 postoperative days, 2 returned to baseline value within 30 days.

N: numbers.

### Subgroups analysis

The aneurysm repair was performed before TF-TAVI in 18/25 (72%) cases in the Simultaneous group and in 9/19 (47%) in the Staged group, performing prior T/EVAR or prior TAVI (Table 4). Overall, 36 (82%) of patients received a EVAR and 8 (18%) a T/EVAR procedure for infrarenal or thoracic aortic pathology, respectively (Table 5).

**Table 4:** Details of the procedures for both Simultaneous and Staged group

	Overall-44, *N* (%)	Simultaneous group			Staged group		
		Overall-25, *N* (%)	EVAR first-18, *N* (%)	TAVI first-7, *N* (%)	Overall-19, *N* (%)	EVAR first-10, *N* (%)	TAVI first–9, *N* (%)
Preoperative factors							
Male	30 (68)	13 (52)	7 (39)	5 (71)	17 (89)	9 (90)	8 (89)
Urgent aneurysm	3 (7)	0 (0)	0 (0)	0 (0)	3 (16)	3 (30)	0 (0)
Hostile bilateral femoral/iliac access	11 (25)	5 (20)	4 (22)	1 (14)	6 (32)	3 (30)	3 (33)
TEVAR	8 (57)	3 (12)	3 (17)	0 (0)	5 (26)	2 (20)	3 (33)
EVAR	36 (43)	22 (88)	15 (83)	7 (100)	14 (74)	8 (80)	6 (67)
Days between procedures (Staged group)					72 (IQR: 57–87)	82 (IQR: 32–86)	65 (IQR: 28–88)
Intraoperative details							
General Anaesthesia TEVAR/EVAR	23 (52)	14 (56)	13 (72)	1 (14)	9 (47)	3 (30)	6 (67)
Percutaneous femoral access TEVAR/EVAR	27 (61)	16 (64)	10 (56)	6 (86)	11 (58)	4 (40)	7 (78)
Percutaneous femoral access TAVI	30 (68)	16 (64)	10 (56)	6 (86)	14 (74)	7 (70)	7 (78)
Need for iliac adjunctive procedures	4 (9)	1 (4)	0 (0)	1 (14)	3 (16)	2 (20)	1 (11)
Technical Success	44 (100)	25 (100)	18 (100)	7 (100)	19 (100)	10 (100)	9 (100)
Postoperative results							
Cardiac adverse events	2 (5)	0 (0)	0 (0)	0 (0)	2 (11)	1 (10)	1 (11)
Cerebrovascular adverse events	2 (5)	1 (4)	0 (0)	1 (14)	1 (5)	0 (0)	1 (11)
Respiratory adverse events	5 (11)	0 (0)	0 (0)	0 (0)	5 (26)	2 (20)	3 (33)
Reinterventions	1 (2)	0 (0)	0 (0)	0 (0)	1 (5)	1 (10)	0 (0)
Vascular access complication	2 (5)	1 (4)	1 (6)	0 (0)	1 (5)	0 (0)	1 (11)
Death	1 (2)	0 (0)	0 (0)	0 (0)	1 (5)	1 (10)	0 (0)

EVAR: endovascular aortic repair; IQR: interquartile range; N: numbers; TAVI: transcatheter aortic valve implantation; TEVAR: thoracic endovascular aortic repair.

**Table 5:** Details of the procedures divided upon aortic repair both as endovascular aortic repair for infrarenal abdominal aorta (EVAR) and thoracic endovascular aortic repair (TEVAR)

	Overall-44, *N* (%)	EVAR-36, *N* (%)	TEVAR-8, *N* (%)
Preoperative factors			
Male	30 (68)	25 (69)	5 (62)
Urgent aneurysm	3 (7)	3 (8)	0 (0)
Hostile bilateral femoral/iliac access	11 (25)	10 (27)	1 (12)
Iliac aneurysm	3 (7)	3 (8)	0 (0)
Simultaneous group	25 (57)	22 (61)	3 (37)
Staged group	19 (43)	14 (39)	5 (62)
Days between procedures (Staged group)	72 (IQR: 57–87)	–	74 (IQR: 58–89)
Intraoperative details			
General anaesthesia TEVAR/EVAR	23 (52)	15 (42)	8 (100)
Percutaneous femoral access TEVAR/EVAR	27 (61)	24 (67)	3 (37)
Percutaneous femoral access TAVI	30 (68)	26 (72)	4 (60)
Need for iliac adjunctive procedures	4 (9)	4 (11)	0 (0)
Technical success	44 (100)	36 (100)	8 (100)
Postoperative results			
Cardiac adverse events	2 (5)	1 (3)	1 (12)
Cerebrovascular adverse events	2 (5)	1 (3)	1 (12)
Respiratory adverse events	5 (11)	2 (6)	3 (37)
Reinterventions	1 (2)	1 (3)	0 (0)
Vascular access complication	2 (5)	2 (6)	0 (0)
Death	1 (2)	0 (0)	1 (12)

IQR: interquartile range.

### Follow-up results

**Figure 1:**
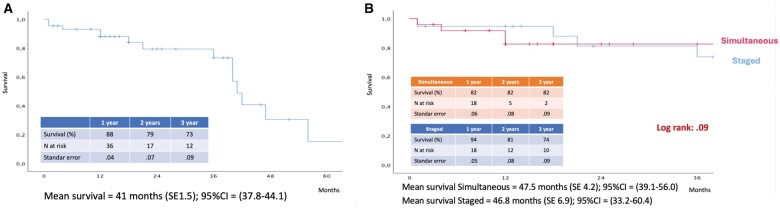
(**A**) Estimated overall survival by Kaplan–Meier analysis. (**B**) Estimated survival by Kaplan–Meier analysis in patients managed by simultaneous and staged approaches.

The median follow-up was 25 (IQR: 8–42) months. Estimated 3-year survival was 73% at Kaplan–Meier analysis, with no difference between groups (Simultaneous: 82% versus Staged: 74%; Log-Rank-*P* = 0.90; Fig. 1). Causes of mortality are summarized in Supplementary Material, Table S2. There was no difference in rehospitalization (Simultaneous: 5/25 versus Staged: 4/19; *P* = 0.30) and procedure-related reinterventions (Simultaneous: 1/25 versus Staged: 2/19; *P* = 1). Causes of rehospitalization and reinterventions are reported in Supplementary Material, Table S3. Two patients underwent reintervention due to iliac recoil after stenting and femoral pseudoaneurysm after percutaneous access.

### Univariate and multivariate analysis

Among primary end-points, staged repair appeared to be a risk factor for pulmonary adverse events [odds ratio = 7.4; 95% confidence interval 3.4–7.6; *P* = 0.006]. Multivariate analysis adjusted for potential confounders confirmed the independent role of the staged procedure (odds ratio = 15.2; 95% confidence interval 5.4–8.9; *P* ≤ 0.001). Follow-up survival was not impacted by staged versus simultaneous approach. The need for permanent cardiac pacemaker was the unique independent factor for follow-up mortality (hazard ratio = 6.3; 95% confidence interval 3.4–7.6; *P* = 0.012).

## DISCUSSION

In the present manuscript, we report 44 patients with concomitant severe AS and T/AAAs, gathered from a multicentre nationwide experience within 7 years. Overall results were satisfactory in terms of technical success, early clinical results and a low number of vascular access complications. Follow-up mortality was also encouraging, especially if we consider high surgical-risk patients.

Concomitant AS and T/AAAs is not uncommon nowadays because of the increasing age of the population as well as multiple aortic comorbidities [1, 7, 13]. Until today, there are no definitive recommendations about the concomitant management of these diseases [1, 7, 8, 13].

Historically, in low-risk patients, the gold standard approach is surgical aortic valve replacement (SAVR) 1st performed by cardiac surgeons followed by aneurysm repair [1, 9]. However, SAVR is usually associated with postoperative increase of systolic blood pressure and risk of aneurysm rupture [14]. On the other hand, issues arise when performing an aneurysm repair as the 1st step due to severe fluctuation of blood pressure during aortic clamping [15].

In the last decades, the endovascular revolutions in both cardiac and vascular surgery allowed to guarantee mini-invasive solutions with effective and reproducible outcomes both for AS and T/AAAs [1, 7]. For these reasons, the current management of concomitant symptomatic and severe AS and T/AAAs is changing, and simultaneous endovascular repair could be feasible.

The 1st report of simultaneous TF-TAVI and EVAR was managed by Drury-Smith *et al.* in 2012 [16]. Table 6 provides a summary of the 25 cases reported in the literature about simultaneous TF-TAVI and EVAR procedures. Bramucci *et al.* [13] reported in 2023 the 1st case of simultaneous TF-TAVI and EVAR performed by total percutaneous approach under local anaesthesia.

**Table 6:** Literature data about simultaneous TF-TAVI and EVAR

Author	Year	Cases	VAC (*n*)	30-day mortality (*n*)	Hospitalization (days)	Follow-up (months)
Naoum	2023	6	2	0	8	19
Bramucci	2023	1	1	0	5	2
Yammine	2021	5	0	0	5	12
Koutsias	2020	2	0	0	9	18
Mauri	2019	2	1	0	10	9
Sato	2017	1	0	0	8	6
Kawashima	2016	1	0	–	9	–
Koudoumas	2015	1	0	0	3	3
Binder	2015	1	0	0	–	3
Aluko	2015	1	0	0	3	12
Marchi	2014	1	0	–	3	–
Chakraborty	2013	1	1	0	–	–
Smith	2012	1	0	0	5	–
Smith	2012	1	0	0	14	6
Overall		25	5	0	7	9
Present series	2023	25	1	0	7	25

EVAR: endovascular aortic repair; TF-TAVI: trans-femoral transcatheter aortic valve implantation; VAC: vascular access complications.

In the present series, we have reported a wide series on this topic and compared cases treated in a single simultaneous procedure with cases managed by staged strategy. Preoperative clinical features were comparable between 2 groups, except for female gender, and more frequent in the Simultaneous group [17].

Even if concomitant TF-TAVI and T/EVAR may increase the complexity of a single procedure, our series demonstrates no differences in intraoperative figures as well as in postoperative mortality between groups. Specifically, postoperative pulmonary adverse events, the need of permanent cardiac pacemaker and length of stay resulted higher in the Staged group. We might speculate to address these findings with the need of multiple hospitalizations, especially in such a fragile population. Moreover, as resulted in the multivariate analysis as collateral finding, the permanent cardiac pacemaker was linked to a reduction in survival during follow-up.

Postoperative AKI is one of the most frequent complications after both T/EVAR and TAVI [18]. Tailored preoperative planning, automated CO_2_ angiography [19] and IVUS (intravascular ultrasound) play a crucial role in the reduction of renal toxicity guaranteeing non necessity of postoperative haemodialysis.

Follow-up results are currently lacking in literature because there are only few preliminary reports describing the feasibility/effectiveness [9, 13, 15, 20, 21]. In the present series, follow-up mortality is not negligible, but acceptable in consideration of the fragile patients’ population.

However, there are still open questions about timing/management, even in the case of concomitant TF-TAVI and T/EVAR. In the present series, numbers are too small to find any statistical association between preoperative morphological/clinical features and different timing of repair. Moreover, the retrospective and multicentre case enrolment plays a role in the heterogeneity of these different approaches, and since every multidisciplinary team based decision on the on specific patients fitness, urgency of the repair per each pathology and institutional protocols, no clear data on the indications are specified in this study, focusing on the procedural aspects. Future researches should better investigate specific morphological/clinical factors that may benefit for a staged or simultaneous approach, given the favourable results from this 1st experience.

### Limitations

The present study has several design limits. It is a retrospective analysis, with small sample size and limited follow-up, with few events ranging from 0 to 5, so type 2 statistical error is to be taken into account, and complex statistical consideration should be considered in light of this.

Eventually, the retrospective design and the inclusion criteria, which were specifically considered just on patients who underwent both procedures, led us to have no data about patients managed by staged approach but unable to complete due to inter-procedural complications mortality.

The main advantage of a concomitant endovascular treatment of both AS and T/AAAs consists of using the same access for both procedures: both EVAR and TAVI require large femoral bore and a combination of both procedures may reduce the risk of vascular access complications, as suggested by the low numbers reported in our cohort, thanks to active hostile iliac vessel preparation [3]. Moreover, it allows to solve in a single procedure 2 different serious illness, avoiding any risk of mortality between therapeutic steps. At the same time, the combined procedure allows to face directly serious related complications: (i) the haemodynamic issues that might be relevant during aortic repair and may be highlighted due to the severe AS; (ii) the risk for aortic rupture or dissection that can arise while navigating TAVI in an aneurysmatic aorta that may suggest to use an alternative approach, such as transapical or axillary ones, none presented in our series of 100% TF-TAVI. (iii) Eventually, a simultaneous approach may also reduce the overall periprocedural costs due to a reduced pulmonary complication rate and shorter hospitalization period.

## CONCLUSIONS

Simultaneous or staged thoracic/abdominal endovascular aortic repair and TAVI are effective with satisfactory outcomes with both strategies. Despite small numbers, simultaneous endovascular repair seems to offer significant reduction of overall hospitalization and pulmonary complications, while maintaining similar procedure-related follow-up outcomes. These data may be considered in the implementation of multidisciplinary teams of Cardiac, Vascular Surgeons and Interventional Cardiologists while evaluating high surgical risk patients presenting both pathologies.

## Supplementary Material

ezae379_Supplementary_Data

## Data Availability

Data were retrospectively collected in each centre from clinical records, shared anonymously and analysed. Due to its retrospective nature, individual informed consent was waived and Institutional Review Board approval was obtained in accordance with the Strengthening the Reporting of Observational Studies in Epidemiology (STROBE) guidelines for the observational studies. All relevant data are within the manuscript and its Supporting Information files. The data of this study are available from the corresponding author upon reasonable request to the corresponding author: DOI:10.5281/zenodo.13997887.

## Notes

Presented at the 37th ESVS Annual Meeting, Belfast, UK, 26–29 September 2023.

## ABBREVIATIONS

AAA: Abdominal aortic aneurysm
AS: Aortic valve stenosis
EVAR: Endovascular aortic repair
IQR: Interquartile range
SAVR: Surgical aortic valve replacement
T/AAA: Thoracic abdominal aortic aneurysm
TAVI: Transcatheter aortic valve implantation
T/EVAR: Thoracic endovascular aortic repair
TF: Trans-femoral
VARC: Valve Academic Research Consortium
