# Supplementary material for: Simultaneous versus staged approach in transcatheter aortic valve implantation for severe stenosis and endovascular aortic repair for thoracic and abdominal aortic aneurysm
Source: Eur J Cardiothorac Surg. 2024 Oct 23;66(5):ezae379. doi: 10.1093/ejcts/ezae379 (PMC11534088; doi:10.1093/ejcts/ezae379)
Supplement: ezae379_Supplementary_Data [file ezae379_supplementary_data.docx]

SUPPLEMENTARY MATERIALS

**Supplementary Table 1.** Details of type of aortic and valve endovascular repair by manufacturer and access size.

| **EVAR + TAVI PATIENTS** | | | | | | |
| --- | --- | --- | --- | --- | --- | --- |
| **Type of EVAR** | **Overall EVAR**  N (%) | | **Access Size**  Fr (Range) | **Type of TAVI** | **Overall TAVI** | **Access Size**  Fr (Range) |
| Overall | 36 (100) | |  | Overall | 36 (100) |  |
| Gore Excluder  Medtronic Endurant  Cook Alpha | 14 (39)  9 (25)  6 (17) | | 16-24  18-20  16-22 | Edwards Sapien 3  Medtronic Evolut Pro  Medtronic Corevalve | 18 (50)  11 (31)  5 (15) | 14-16  14  16-18 |
| Endologix (AFX/Alto) | 5 (14) | | 17 |  |  |  |
| Cordis Incraft | 2 (6) | | 14 |  |  |  |
| **TEVAR + TAVI PATIENTS** | | | | | | |
| **Type of TEVAR** | **Overall TEVAR**  N (%) | **Access Size**  Fr (Range) | | **Type of TAVI** | **Overall TAVI** | **Access Size**  Fr (Range) |
| Overall | 8 (100) |  | | Overall | 8 (100) |  |
| Cook Alpha | 5 (62) | 18-22 | | Edwards Sapien 3 | 4 (50) | 14-18 |
| Gore TAG | 2 (25) | 22-24 | | Medtronic Evolut Pro | 2 (25) | 14 |
| Bolton Relay | 1 (13) | 24 | | Medtronic Corevalve | 1 (12) | 14 |
|  |  |  | | MyVal | 1 (13) | 14 |

**Supplementary Table 2.** Causes of mortality at long term follow up

|  | **Overall**  N (%) | **Simultaneous**  N (%) | Staged  N (%) |
| --- | --- | --- | --- |
| Overall | 14 (100) | 6 (43) | 8 (57) |
| Cancer | 2 (14) | 1 | 1 |
| Cardiac | 2 (14) | 1 | 1 |
| Covid 19 infection | 3 (22) | 1 | 2 |
| Respiratory | 2 (14) | 1 | 1 |
| Infection / Multiorgan failure | 2 (14) | 0 | 2 |
| Unknow | 3 (22) | 2 | 1 |

**Supplementary Table 3.** Causes of readmission and reinterventions after 30 postoperative days

|  | **Overall -** 44  N (%) | **Simultaneous -**25  N (%) | **Staged -**19  N (%) |
| --- | --- | --- | --- |
| Reinterventions | 2 (5) | 1 (4) | 1 (5) |
| Readmission | 9 (20) | 5 (20) | 4 (21) |
| Procedure related  Superficial femoral artery pseudoaneurysm  Iliac stent re-stenosis  Aortic endograft infection | 3  1  1  1 | 1  -  1  - | 2  1  -  1 |
| Procedure unrelated  Cardiac infarct  Umbilical hernia  Covid infection  Cerebral infection | 6  2  1  2  1 | 4  1  1  1  1 | 2  1  -  1  - |
